# Association of Tumor Growth Factor-β and Interferon-γ Serum Levels with Insulin Resistance in Normal Pregnancy

**DOI:** 10.5539/gjhs.v8n6p25

**Published:** 2015-09-28

**Authors:** Abdolreza Sotoodeh Jahromi, Mohammad Sadegh Sanie, Alireza Yusefi, Hassan Zabetian, Parvin Zareian, Hossein Hakimelahi, Abdolhossien Madani, Mohammad Hojjat-Farsangi

**Affiliations:** 1 Research Center for Non-Communicable Diseases, Jahrom University of Medical Sciences, Jahrom, Iran; 2 Research center for social determinants of health, Jahrom University of Medical Sciences, Jahrom, Iran; 3 Research Center for Social Determinants in Health Promotion, Hormozgan University of Medical Sciences, Bandarabbas, Iran; 4 Department of Oncology-Pathology, Immune and Gene therapy Lab, Cancer Center Karolinska (CCK), Karolinska University Hospital Solna and Karolinska Institute, Stockholm, Sweden

**Keywords:** IFN-γ, TGF-β, Insulin resistance, normal pregnancy

## Abstract

Pregnancy is related to change in glucose metabolism and insulin production. The aim of our study was to determine the association of serum IFN-γ and TGF-β levels with insulin resistance during normal pregnancy. This cross sectional study was carried out on 97 healthy pregnant (in different trimesters) and 28 healthy non-pregnant women. Serum TGF-β and IFN-γ level were measured by ELISA method. Pregnant women had high level TGF-β and low level IFN-γ as compared non-pregnant women. Maternal serum TGF-β concentration significantly increased in third trimester as compared first and second trimester of pregnancy. Maternal serum IFN-γ concentration significantly decreased in third trimester as compared first and second trimester of pregnancy. Pregnant women exhibited higher score of HOMA IR as compared non-pregnant women. There were association between gestational age with body mass index (r=0.28, P=0.005), TGF-β (r=0.45, P<0.001) and IFN-γ (r=-0.50, P<0.001). There was significant association between Insulin resistance and TGF-β (r=0.17, p=0.05). Our findings suggest that changes in maternal cytokine level in healthy pregnant women were anti-inflammatory. Furthermore, Tumor Growth Factor-β appears has a role in induction insulin resistance in healthy pregnant women. However, further studies needed to evaluate role of different cytokines on insulin resistance in normal pregnancy.

## 1. Introduction

Pregnancy is related to change in glucose metabolism and insulin production, regulation and action. During normal pregnancy an obvious physiological decline in peripheral insulin sensitivity occurs as pregnancy proceeds (Kirwan, Hauguel-De Mouzon et al., 2002). However, the mechanism responsible for insulin resistance (IR) has not been stated clearly. There are reports that show longitudinal changes in insulin resistance in healthy and diabetic subjects have been correlated with cytokines and adipokine concentration (Bruun, Stallknecht, Helge, & Richelsen, 2007; Fève & Bastard, 2009). Cytokines and adipokines are important messengers of activation in the immune system (Fantuzzi, 2005).

During normal pregnancy, the maternal immune system and cytokine profile alter to prevent the rejection of the fetal allograft and preservation of pregnancy (Szarka, Rigó, Lázár, Bekő, & Molvarec, 2010; Jahromi, Zareian, & Madani, 2011a, 2011b; Jahromi, Shojaei, & Ghobadifar, 2014). In addition previous studies have been shown, there is correlation between insulin resistance with maternal level of a number of cytokines in normal and complicated pregnancies (Zhang et al., 2006). IFN-γ is a cytokine that secrets by Th1cells. It has role in the regulation of almost all phases of immune and inflammatory responses. This cytokine was identified in all gestational tissues (Veith & Rice, 1999). IFN-γ mediated signaling, is essential for normal maintenance of maternal tissues in implantation sites during mid gestation (Ashkar & Croy, 1999). There are studies concerning its role in the pathogenesis of insulin resistance. IFN-γ attenuates insulin sensitivity and reduced lipid storage in human adipocytes (McGillicuddy et al., 2009) and has a role in regulating systemic inflammation and insulin resistance in obesity (O’Rourke, White, Metcalf, Winters, Diggs, Zhu, & Marks, 2012). Injection natural human leukocyte IFN-α twice overnight in eight healthy subjects caused mild influenza like symptoms and induced a rise in circulating glucose and insulin (Koivisto, Pelkonen, & Cantell, 1989).

Transforming growth factor beta (TGF-β) is another type of cytokines that exhibits potent immunoregulatory and anti-inflammatory properties. It secretes by many cell types, including macrophages. In pregnancy period it is secreted by endometrium and placenta (Jones, Stoikos, Findlay, & Salamonsen, 2006). This type of cytokine correlates with obesity induced insulin resistance (Romano et al., 2003) and TGF-Β-β/Smad3 signaling pathway plays key roles in development of insulin resistance in genetically obese mice (Tsurutani et al., 2011).

However, the most of previous studies have measured IFN-γ and TGF-β - levels in human placenta or fetal tissues (Gruppuso et al., 1991; Paulesu et al., 1994; Xuan et al., 2007) and only, a few of studies have reported maternal circulating levels of IFN-γ and TGF-β at different stages of pregnancy (Kraus et al., 2010; Singh et al., 2013). Additionally, at the time 0f present study, there was no reports have assessed the association of TGF-β - and IFN-γ level with IR during normal pregnancy. Therefore, the aim of present study was to determine the association of serum IFN-γ and TGF-β levels with insulin resistance.

## 2. Material and Methods

This cross sectional study was done at the department of Obstetrics and Gynecology of Honary Clinic, Jahrom, Iran, autumn, 2013 to the spring, 2014. Subjects were 97 pregnant women with different gestational ages (first trimester: 32, second trimester: 25, third trimester: 40) and 28 non-pregnant women similar in age and body mass index. The Subjects with clinical conditions such as diabetes, hypertension, autoimmune disease and other illness were excluded from the study. None of the women was on any drug therapy.

Maternal height and pregnancy weight were measured in women and BMI was calculated according to these measurements. Blood samples were taken after 8 hours fasting time, then immediately the serum was separated by centrifugation at 2,500 rpm (10 minutes). The samples were processed directly or in a week following preservation at -70°C. Glucose measurements (intra-assay coefficient of variation, 2.1%, inter-assay CV 2.6%) were done using the glucose oxidize method. Insulin level was measured by ELISA technique using commercial kit (IBL-IB79167). Insulin resistance was determined using Homeostatic Model Assessment (HOMA-IR) index equation (Johnson, 2008). Serum TGF-β was measured by commercial ELISA kit (Bender Med Systems, Austria: Cat. No. BMS249/4) followed company instruction. Serum IFN-γ was measured by ELISA (Bender Med Systems, Austria: Cat. No. BMS228), According to company instruction as the same method for serum TGF-β. The ethical committee of Jahrom University of Medical Sciences approved the study. All women in this study filled and signed informed consent letter.

Statistical analysis: All values were displayed as Mean ± SEM. Data were tested for normal distribution using the Kolmogorov–Smirnov test. One way ANOVA analysis and Post hoc test (LSD test) were used for comparing mean among the groups and correlations were calculated using liner correlation (Pearson). SPSS software (version 11.5) was used for data analysis. p-value of < 0.05 was considered statistically significant.

## 3. Results

The clinical characteristics and laboratory findings of the participants are described in Table 1.

**Table 1:** Clinical and laboratory characteristics of pregnant and non-pregnant women

	control	Pregnant women
Number of case	28	**97**
age (years)	27.4±1	**26.2±0.4**
BMI (Kg/m^2^)	23.3±0.5	**25.3±0.4**
Fasting blood sugar (mg/dl)	80.0 ± 9.2	**79.4± 9.7**
Insulin level (µLU/ml)	9.2±1.95	10.6±3.3**
TGF-β (pg/ml)	35.26±6.02	40.15±6.38**
Insulin resistance	1.77±0.41	2.06±0.66**
IFN-γ pg/ml	2.19±1.91	**1.60±0.9**

** P< 0.01(control).

**Figure 1:**
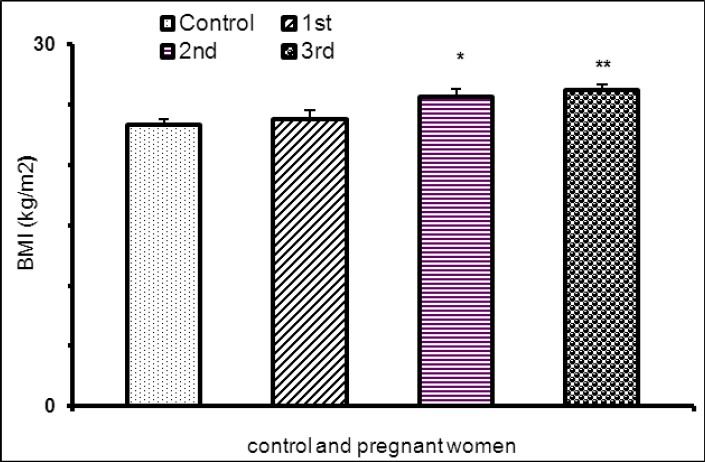
BMI in pregnant women in tri-trimester pregnancy and non pregnat women (*P<0.05, **P<0.01)

Body mass index significantly was higher in pregnant women in second and third trimester as compared with non –pregnant and women in first trimester of pregnancy Figure 1, Table 2.

**Table 2:** Clinical and laboratory characteristics of pregnant women in three trimesters

	1^st^ trimester	2^nd^ trimester	3^rd^ trimester
Number of case	32	25	**40**
age (years)	25.1 ±0.7	25.2±0.4	27.5±0.7*ψ
BMI (Kg/m2)	23.8±0.7	25.6±0.7*	26.2±0.5***
Fasting blood sugar(mg/dl)	79.2±1.6	78±1.9	**80.4±1.8**
Insulin level (µlU/ml)	10.60±0.52	11.38±0.67	**10.21±0.55**
TGF (pg/ml)	36.7 ± 1.2	39.6 ± 1.2	43.5 ± 0.64****
Insulin resistance	2.0± 0.1	2.1 ± 0.12	**2 ± 0.11**
IFN-γ (pg/ml)	2.09 ± 0.13	1.93 ± 0.18	0.99± 0.09 ****

* P=0.05 (first trimester),

*** P< 0.001(first trimester),

**** P<0.01 (second trimester),

* ψ P<0.05 (second trimester).

Score of HOMA IR was higher in pregnant women as compared non-pregnant women but there was no statistical difference in this score among pregnant women (Tables 1 and 2).

**Figure 2:**
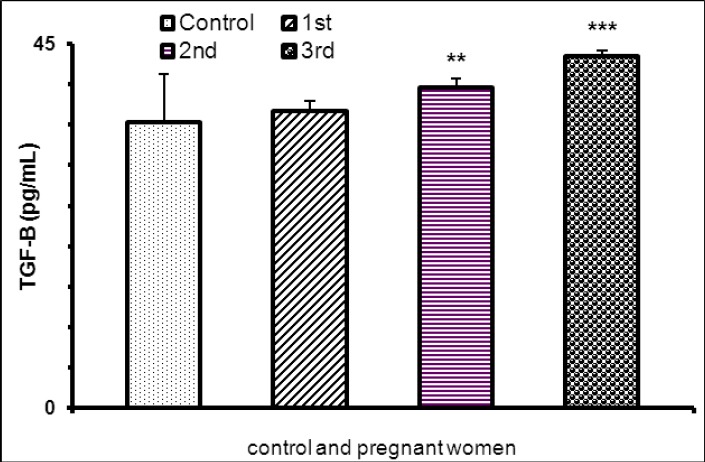
TGF-B concentration in pregnant and non-pregnant women (**P<0.01, ***P<0.001)

Pregnant women had also high level TGF-β as compared non-pregnant women Table 1. Maternal serum TGF-β concentration was higher in third trimester as compared first and second trimester of pregnancy (Table 2). In addition, pregnant subjects with gestational age more than 12 weeks had higher TGF-β level as compared healthy non-pregnant women (Figure 2).

**Figure 3:**
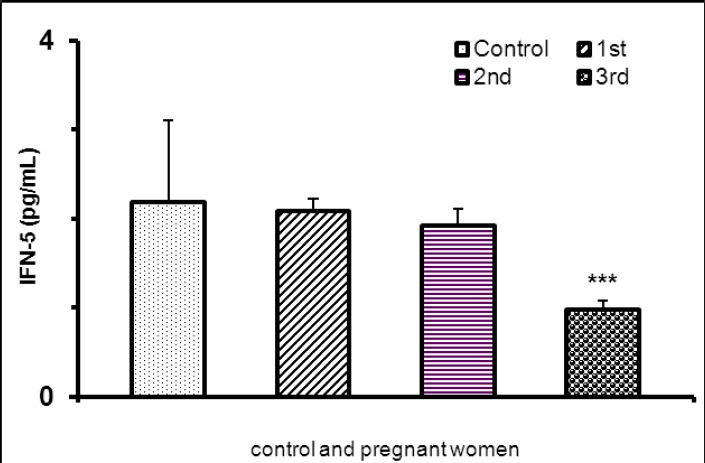
IFN-gamma concentration in pregnant and non-pregnant women (***P<0.001)

In pregnant women, IFN-γ significantly was lower as compared non-pregnant women (Table 1). Maternal serum IFN-γ concentration significantly decreased in 3rd trimester as compared first and second trimester of pregnancy (Table 2), but there were not statistical difference in serum IFN-γ concentration between the non –pregnant women and pregnant women in first and second trimester of pregnancy (Figure 3).

The gestational age was positively correlated with BMI (r= 0.28, P=0. 005) and TGF-β (r=0. 45, P<0. 001) and was negatively correlated with IFN-γ (r= -0. 5, P<0. 001).

Insulin resistance were not related to gestational age(r=-0. 12, P≥0. 200) and IFN-γ level (r=-0. 12, P≥0. 100).

There was not significant correlation between Insulin resistance with gestational age (r=-0.12, P≥0. 200) and IFN-γ level (r=-0. 12, P≥0. 100) but there was positive significant relation between Insulin resistance and TGF-β (r=0.17, P≥0.05).

The significant negative association was also between IFN-γ concentration with BMI (r= -0. 2, P=0.01).

## 4. Discussion

In this study, we determined insulin resistance, maternal circulating levels of TGF-β and IFN-γ in healthy non-pregnant and pregnant women.

During normal pregnancy glucose metabolism and insulin production alters and results to insulin resistance (Johnson, 2008). Our data confirm previous results that insulin resistance was increased in pregnant patients (Kirwan et al., 2002). In present study, the values of TGF-β in the serum of pregnant women were higher than those in healthy control women suggesting that TGF-β1levels rise during pregnancy. TGF-β isoforms involved in the control of apoptosis in the uterus during pregnancy (Shooner et al., 2005) and apoptosis plays an important role during embryo implantation and during late pregnancy, especially during regression of the decidua basalis (Welsh, 1993; Pampfer & Donnay, 1999). It seems TGF-B regulates trophoblast invasion and proliferation and may function as a regulatory factor in fetal allograft survival during pregnancy (Slater & Murphy, 2000; Ayatollahi et al., 2005). Contrary to our findings of an increase in serum level TGF-β with rise in gestational age, Mandeep sigh et al. reported maternal serum concentration fell during pregnancy from 52.7 ± 5.5 ng/ml at 10-week to 46.8 ± 5.5 ng/ml at 20-week pregnancy and to 40.5 ± 3.8 ng/ml at 26-week pregnancy. The main difference between the two studies was difference between assay techniques used in two studies and ethnicity (Singh et al., 2013). In this work, we found positive correlation between IR with TGF-β. Huei-Min Lin et al. have been shown that TGF-β/Smad3 signaling is an important regulator of insulin gene transcription and β-cell function and TGF-β signaling represses insulin gene transcription (Lin et al., 2009). Also TGF-β signaling regulates glucose tolerance (Yadav et al., 2011). In our study, IFN-γ level was lower in pregnant women compared non-pregnant women and a significant negative correlation were obtained between IFN-γ level and gestational age.

The predominance of Th2-cell derived cytokines over Th1 cell derived cytokines have been demonstrated in normal pregnancy (Marzi et al., 1996). Previous studies about maternal IFN-γ during normal pregnancy have produced conflicting results with some showing increased (Curry et al., 2008) and others showing decreased level IFN-γ (Paulesu et al., 1994; Veith & Rice, 1999; Bates et al., 2002). IFN-γ has important role in initiation of endometrial vasculature remodeling, angiogenesis at implantation sites and preservation of the decidual part of the placenta. In humans, deviations in these processes result to serious gestational Problems, such as fetal loss or preeclampsia (Brakhas et al., n. d.; Murphy et al., 2009). In addition, IFN-γ mediated activation of major histocompatibility complex (MHC) molecules on trophoblast cells. During normal pregnancy, there is intrinsic regulatory mechanism that prevents IFN-γ induced expression of MHC molecules and transplant rejection reactions by maternal lymphocytes. Therefore results to successful pregnancy.

However, gestational difficulties such as fetal death have been related to increase in IFN-γ (Germain et al., 2007). The results of this study did not show correlation between IFN-γ and IR, but there was significant negative correlation between IFN-γ and BMI in pregnant subjects. In other word, with increase in gestational age and body mass index, serum level IFN-γ decreases. A study reported the significant negative correlation between IFN-γ and BMI in healthy subjects (Zheng et al., 2013). In contrast, other human studies showed higher level IFN-γ in overweight and obese subjects than normal weight subjects (Pacifico et al., 2006; Utsal et al., 2012).

In this study, alteration in maternal cytokine level in healthy pregnant women can be regarded as an anti-inflammatory response as shown by the decreased inflammatory cytokine (IFN-γ) and increased anti-inflammatory cytokine (TGF-β) concentration. In addition, TGF-β was positively associated with insulin resistance and gestational age.
